# Editorial: Economic evaluation of mental health interventions

**DOI:** 10.3389/fpsyt.2023.1294245

**Published:** 2023-09-28

**Authors:** Huajie Jin, Ali Jalali, Ben Wijnen, Yuhua Bao

**Affiliations:** ^1^King's Health Economics (KHE), Institute of Psychiatry, Psychology and Neuroscience at King's College London, London, United Kingdom; ^2^Department of Population Health Sciences, Weill Cornell Medicine, New York, NY, United States; ^3^Center for Economic Evaluations, Trimbos Institute, Utrecht, Netherlands

**Keywords:** economic evaluation, editorial, mental health, cost-effectiveness analysis, cost-of-illness

## Introduction

Mental health disorders affected more than 1 billion people globally and were responsible for 7% of the global burden of disease as measured in Disability Adjusted Life Years (DALYs) and 19% of all years lived with disability (1). Economic evaluation is increasingly employed to guide resource allocation decisions and policymaking in mental health.

Our Research Topic showcases cutting-edge economic evaluations in mental health to inform public and private resource decisions. It includes nine papers: two partial economic evaluations focusing solely on the costs of interventions/diseases, three full economic evaluations assessing both costs and consequences of interventions, two systematic reviews, one perspective paper, and one protocol paper.

## Partial economic evaluations

Based on data from two RCTs, Paterson et al. estimated that delivering a mental health recovery narrative web application costs £349 per user for those with psychosis and £241 per user for those without psychosis. Crucially, this study accounts for intervention development costs, which is important but often overlooked in costing studies. The results of this study can be used to estimate the cost of delivering NEON at scale and improve consistency in reporting of cost for similar digital health interventions.

Based on multiple sources of information, Sousa et al. estimates the total disease burden of Treatment-Resistant Depression and Major Depression with Suicide Risk in Portugal at 66.3 thousand DALYs. Direct costs were €30.8 million, mainly from medical appointments and medication. Adding productivity losses, the total cost reached €1.1 billion. This study emphasized the need for prioritizing health promotions for both disorders.

## Full economic evaluations

Of the three full economic evaluations included in this topic, Le Novere et al. conducted a trial-based economic evaluation, in which the trial provides the main source of input data; while Liu et al.(a) and Kleijburg et al. employed a decision-analytic modeling approach.

Le Novere et al. assessed the cost-effectiveness of peer-supported self-management for people discharged from mental health crisis teams in England, from a mental health service perspective. Compared to usual care, the intervention had a 57% chance of being cost-effective at £20,000 per QALY gained. The main methodological challenge in this study is the significant (nearly 50%) missing data for the utility outcome, while data regarding resource use were nearly complete. This study illustrates how common strategies to address missingness or distributional features of cost and utility data may or may not mitigate biases.

Both Liu et al.(a) and Kleijburg et al. employed a Markov modeling approach, dividing the disease into distinct states with assigned transition probabilities for movement over discrete time periods, known as “Markov cycles” (2). By attaching costs and health outcomes to each of these states and using intervention-specific transition probabilities, a Markov model can be used to estimate the long-term costs and outcomes for the interventions of interest.

Using a previously published model, Liu et al.(b) found that lurasidone was a dominant treatment compared to olanzapine and risperidone in the first-line treatment of schizophrenia in China, resulting in greater QALY gains at lower costs. Kleijburg et al. reported the development of TiBipoMod—A model which can simulates the lifetime costs and health outcomes for various interventions in the treatment of bipolar disorders type I and II, from a societal perspective. A case study conducted based on TiBipoMod showed that mindfulness-based cognitive therapy dominates standard care.

## Systematic reviews

Systematic reviews of economic evaluations of healthcare programs and interventions can synthesize crucial data to inform healthcare decision-making and highlight research priorities (3). However, synthesizing evidence from such studies is challenging given inconsistencies in cost-effectiveness research designs (e.g., synthesizing evidence from simulation and trial-based analyses) and reporting guidelines (4, 5). The two systematic reviews included in this Research Topic are notable in that they both follow the Consensus on Health Economic Criteria (CHEC) list for assessing the methodological quality of economic evaluations in systematic reviews (6), and both provide a thorough discussion of the limitations in consolidating evidence across the included studies.

Kugener et al. provide a timely update on the economic evidence for prevention and treatment interventions for child maltreatment, abuse and neglect in high-income countries (US, Australia, UK, Canada). Their study evaluated a total of 11 studies, 7 of which were model-based economic evaluations while 4 were conducted alongside a clinical trial. All studies demonstrated improved outcomes at common cost-effectiveness value thresholds, with two demonstrating cost-savings in addition to effectiveness gains. Kugener et al. noted that cross study comparisons and/or pooling was made difficult due to limited comparability of measures across studies, including lack of commonly applied terminology for child maltreatment, as well as variation in the methodological rigor such as hand lined missing data, which continues to be an issue in the field (7).

Hannah et al. reviewed economic evidence pertaining to interventions for treatment-resistant depression. Their review encompassed 31 studies-−11 conducted alongside clinical trials, and 20 used modeling methods. Similar to Kugener et al., Hannah et al. identified heterogeneity in methodological quality, most notably finding that fewer than half of the model-based evaluations conducted a comprehensive sensitivity analysis of model parameters. An important feature of Hannah et al.'s review is their in-depth discussion on the divergences between the model vs. trial approaches to economic evaluations.

## Others

The successful implementation of mental health interventions, particularly behavioral health interventions, frequently demands significant stakeholder engagement. Nonetheless, the expenses associated with this involvement are commonly overlooked in current economic evaluations. In their perspective paper, Raciborski et al. delve into the integration of stakeholder engagement with established economic analysis methods, aiming to enhance decision-making regarding the implementation of behavioral health interventions.

Shah et al. reports on a protocol for a return-on-investment analysis of system-wide service transformation for young people experiencing mental health problems in Canada. Novelties of the proposed study lie in two aspects: economic evaluation of a system transformation (rather than a particular health technology) and assessing population-wide implications of the system intervention (thus capturing complex links between intervention and outcomes and spillovers). Findings of the proposed studies will inform decisions regarding large scale, system transformation initiatives designed to benefit population health.

## Author contributions

HJ: Conceptualization, Data curation, Investigation, Methodology, Writing—original draft, Writing—review and editing. AJ: Conceptualization, Data curation, Investigation, Methodology, Writing—original draft, Writing—review and editing. BW: Conceptualization, Data curation, Investigation, Methodology, Writing—original draft, Writing—review and editing. YB: Conceptualization, Data curation, Investigation, Methodology, Writing—original draft, Writing—review and editing.
